# Supplementary figures and images for: Basking shark sub-surface behaviour revealed by animal-towed cameras
Source: PLoS One. 2021 Jul 28;16(7):e0253388. doi: 10.1371/journal.pone.0253388 (PMC8318306; doi:10.1371/journal.pone.0253388)

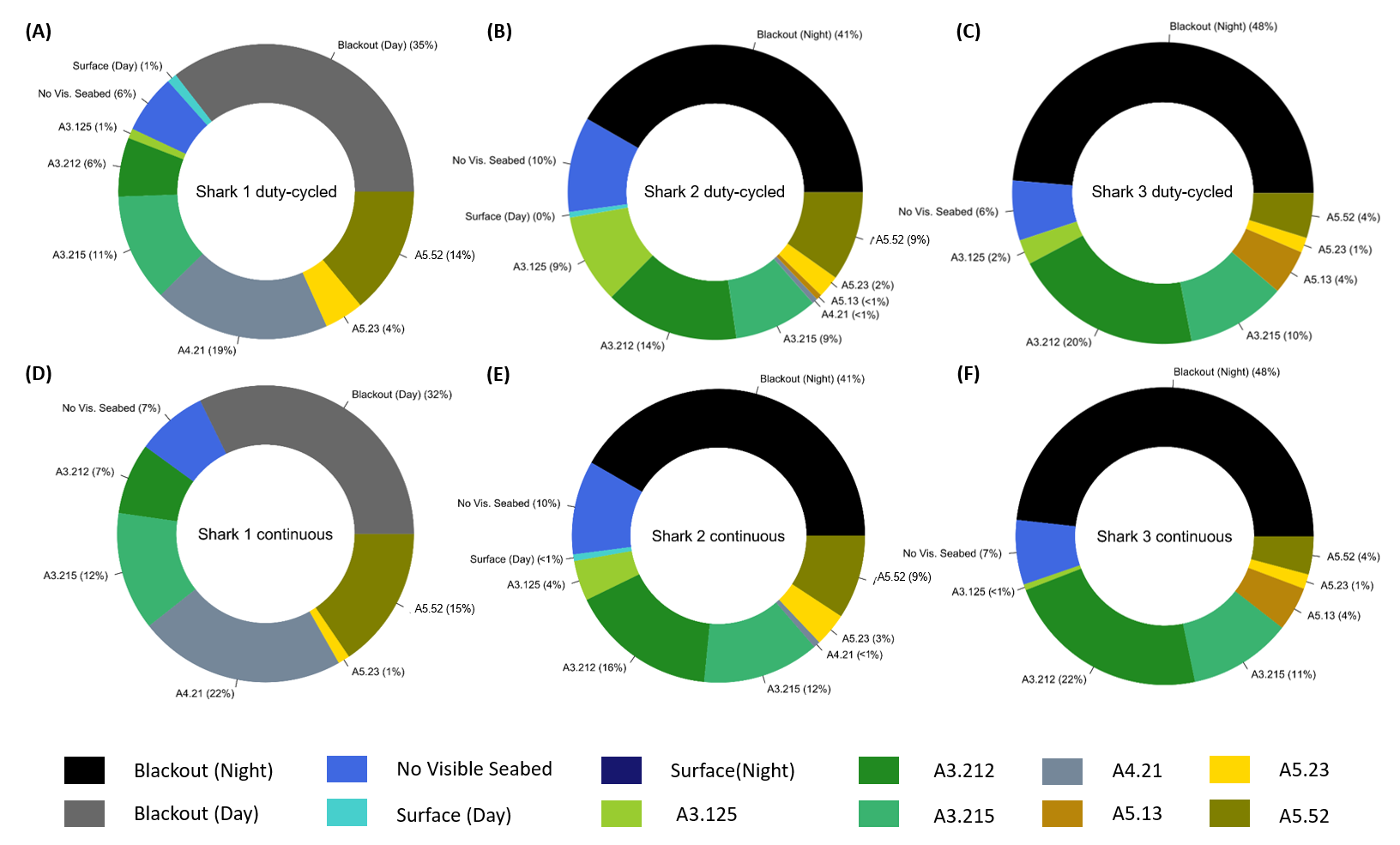

Supplement: S1 Fig — Proportion of time sharks 1–3 spent swimming over different habitat types characterised by their European Nature Information System (EUNIS) code (A3.125 mixed kelp with opportunistic red seaweed on sand-covered infralittoral rock, A3.212 Laminaria hyperborea on tide-swept infralittoral rock, A3.215 Dense foliose red seaweeds on silty moderately exposed infralittoral rock, A4.21 Echinoderms and crustose communities on circalittoral rock, A5.13 Infralittoral coarse sediment, A5.23 Infralittoral fine sand, A5.52 Kelp and seaweed communities on sublittoral sediment) derived from duty-cycled data (A-C) and continuous data (D-F). Includes proportion of time sharks spent in the water column (No Vis. Seabed), or when habitat could not be characterised owing to poor light conditions from deep diving (Blackout Day) or recording at night (Blackout Night). (TIF) [file pone.0253388.s001.tif]
