# Supplementary material for: Basking shark sub-surface behaviour revealed by animal-towed cameras
Source: PLoS One. 2021 Jul 28;16(7):e0253388. doi: 10.1371/journal.pone.0253388 (PMC8318306; doi:10.1371/journal.pone.0253388)
Supplement: S1 Table — (DOCX) [file pone.0253388.s002.docx]

**S1 Table**. Summary table of the European Nature Information System (EUNIS) habitat codes used to classify the habitat types used by the basking sharks.

| **EUNIS Code** | **Description** |
| --- | --- |
| A3.125 | Mixed kelps with scour-tolerant and opportunistic foliose red seaweeds on scoured or sand-covered infralittoral rock |
| A3.212 | *Laminaria hyperborea* on tide-swept, infralittoral rock |
| A3.215 | Dense foliose red seaweeds on silty moderately exposed infralittoral rock |
| A4.21 | Echinoderms and crustose communities on circalittoral rock |
| A5.13 | Infralittoral coarse sediment |
| A5.23 | Infralittoral fine sand |
| A5.52 | Kelp and seaweed communities on sublittoral sediment |
| No vis. seabed | Shark swimming mid-water, where neither the surface or seabed are visible |
| Surface (Day) | Sea surface visible for >50% of clip during daylight hours |
| Surface (Night) | Sea surface visible for >50% of clip during the night |
| Blackout (Day) | Video data too dark to observe habitat, usually due to deep diving, during daylight hours |
| Blackout (Night) | Video data to dark to observe habitat during the night |
