# Supplementary material for: Basking shark sub-surface behaviour revealed by animal-towed cameras
Source: PLoS One. 2021 Jul 28;16(7):e0253388. doi: 10.1371/journal.pone.0253388 (PMC8318306; doi:10.1371/journal.pone.0253388)
Supplement: S2 Table — Statistics for sharks 1 to 3 are derived from sub-sampled video data to match the format of duty cycled tags deployed in 2019. (DOCX) [file pone.0253388.s003.docx]

**S2 Table.** Summary information on deployment of towed camera tags (2018 and 2019), including locations of deployment, tag detachment and retrieval times, attachment and data duration and camera performance. Statistics for sharks 1 to 3 are derived from sub-sampled video data to match the format of duty cycled tags deployed in 2019.

| Name  (sex) | Total length (m) | Deploy date / time  (BST) | Deploy location  (lat, lon) WGS84 | Detach location  (lat,lon)  WGS84 | Release type | Cam end date/ time  (BST) | Cam detach date/ time  (BST) | Max  depth (m) | Attach duration (hours) | Duty-cycled video data duration (hours)  Total  (hours) | No. video data files | No. & (%) video data with blackout  DAY | No. & (%) video data with blackout  NIGHT | No. & (%) video data with strobing | No. & (%) video data with unstable camera |
| --- | --- | --- | --- | --- | --- | --- | --- | --- | --- | --- | --- | --- | --- | --- | --- |
| Shark 1 (F) | 5-6 | 2-Aug-18 1344 | 56.57129 -6.75475 | 56.60501  -7.14981 | Galv. | 2-Aug-18 2048 | 2-Aug-18 2033 | 130.5 | 7.8 | 0.8  (8) | 97 | 33  (35%) | 0  (0%) | 3  (3%) | 62  (67%) |
| Shark 2 (M) | 6-7 | 2-Aug-18 1447 | 56.57273 -6.75305 | 56.42416 -6.88622 | PTR | 3-Aug-18 0319 | 3-Aug-18  1032 | 49.3 | 20.8 | 1.36  (13.6) | 163 | 0  (0%) | 68  (42%) | 5  (3%) | 53  (33%) |
| Shark 3 (F) | 6-7 | 2-Aug-18 1530 | 56.56613  -6.75588 | 56.34316  -6.706542 | PTR | 3-Aug-18 0648 | 3-Aug-18  1217 | 48.4 | 21.8 | 1.57  (16.3) | 188 | 0  (0%) | 91  (48%) | 41  (22%) | 65  (35%) |
| Shark 4 (U) | 5 | 19-Jul-19 1433 | 56.5788  -7.0908 | 58.3439  -6.6671 | PTR | 20-Jul-19  0247 | 24-Jul-19  0704 | 68..2 | 112.52 | 2.45  (12.24) | 294 | 6  (2%) | 108 (37%) | 0  (0%) | 92 (31%) |
| Shark 5 (M) | 6 | 19-Jul-19 1632 | 56.5976  -7.0433 | 56.9201  -6.6865 | PTR | 20-Jul-19  2253 | 24-Jul-19  0738 | 73.3 | 111.10 | 6.08  (30.35) | 730 | 20  (3%) | 150 (21%) | 0 (0%) | 260 (36%) |
| Shark 6 (F) | 6-7 | 19-Jul-19 1755 | 56.5827  -6.7858 | 56.8305  -6.8976 | PTR | 19-Jul-19  1216 | 24-Jul-19  0727 | 58.8 | 109.54 | 8.48  (42.34) | 1018 | 8  (1%) | 320 (31%) | 30 (3%) | 140 (14%) |
