# Supplementary material for: Basking shark sub-surface behaviour revealed by animal-towed cameras
Source: PLoS One. 2021 Jul 28;16(7):e0253388. doi: 10.1371/journal.pone.0253388 (PMC8318306; doi:10.1371/journal.pone.0253388)
Supplement: Attachment — Submitted filename: Response_to_reviewers.docx [file pone.0253388.s004.docx]

|  |  |
| --- | --- |

University of Exeter

College of Life & Environmental Science

Hatherly Laboratories

Prince of Wales Road, Exeter EX4 4PS, UK

E: [l.hawkes@exeter.ac.uk](mailto:l.hawkes@exeter.ac.uk)

Date: 18 May 2021

**Re:** PONE-D-21-03869

Dear Dr Hyrenbach,

Thank you for handling our manuscript: *‘***Basking shark sub-surface behaviour revealed by animal-towed cameras**’. We found the two sets of review comments to be very helpful in improving the work, and we have added an acknowledgement to this effect at the end of the manuscript. We believe we have now addressed all of the comments and have resubmitted the revised manuscript, and detail our responses to the review comments in blue, below.

Changes made to the reference list relate to references [59] Rudd et al. 2021 which has been published since the current manuscript was first submitted, and reference [100] Hooker et al. 2008 which was added in response to Reviewer 1’s first comment.

Amended Funding Statement:

The funder, NatureScot, is an independent government advisory agency rather than a commercial organisation.

“The funder provided support in the form of salaries for authors [S.M.H], but did not have any additional role in the study design, data collection and analysis, decision to publish, or preparation of the manuscript. The specific roles of this author are articulated in the ‘author contributions’ section.”

Updated Competing Interests Statement:

Since the funder, NatureScot, is not a commercial organisation, we do not have any commercial affiliations. This does not alter our adherence to PLOS ONE policies on sharing data and materials.

Yours sincerely

Lucy Hawkes

**Reviewer Comments 1:**

1) Given the relative paucity of studies using cameras, it might be worthwhile to compare your 2018 continuous records to your 2018 subsampled records to match the 2019 duty cycle. In particular, I would focus on the ability to provide representative summary statistics as well as any potential quantitative comparisons between dive profiles. This would have a high value added effect for the field of biologging and push the study (or subsequent publications) into an area with utility for biologging as a whole.

We have now done this, and added it to the revised manuscript, see track changes through the methods (line 159), results (lines 243-248, line 273, and line 424-438). We’ve also added some discussion about the differences at line 556-575.

2) Can you use the camera to interpolate behaviors in the “black-out” period? I would think that this a real boon to the use of cameras over accelerometers/depth recorders alone. In particular, we know that basking sharks can exhibit some strong diel vertical migratory behavior, especially in UK waters, so this might be particularly useful in this region.

Unfortunately, the camera did not have a lighting system (which would have drawn on the battery, reduced the recording duration and likely introduce behavioural modification in either the tagged animals or conspecifics), so at depth the footage was totally blacked out. We’ve tried to make this clearer by adding a statement about a lack of lighting to the methods. Thus, no behaviour can be inferred from this video footage except to validate that the sharks have swam to depth and deeper than the capabilities / light sensitivity of the camera, which would be identified from time-depth recorders, or that it is night time.

3) **Minor corrections**:
   Line 19: Reads: “Basking sharks second large shark species in the world” maybe change to “Basking sharks, the second largest….”

Changes made as suggested

**Reviewer 2 Comments**:

1) L67-70. These two sentences are a bit repetitive. The first says "Animal-borne cameras can also document [...]" and the second "Cameras may also identify possible [...]." Perhaps the authors could merge both in a succinct way?

Sentences now merged to reduce repetition.

2) L173. Methods are quite well explained, except in L173 where it says "[...] (TBF) was used as a measure of behavioural state". It is not clear to me to what behavioural states the authors are refereeing to. Cannot seem to find that information. Please define which states are these and how they were classified.

Deleted “measure if behavioural state” to remove ambiguity of the term.

3) L180. The sentence defining the feeding behaviour should be moved to near the L167, when other behaviours are being referred to.

Changes made as suggested, the sentence was incorporated in L167.

4) L253. Figures are generally quite nice. But they should stand alone. The information of the EUNIS seabed codes should be included in the figure too, as a legend. Referring that information to Table S1 is not good enough.

Descriptions of the EUNIS codes have been included in the legend of figure 4 and no longer references Table S1 for more information.
